# Idiopathic intracranial hypertension without papilledema in chronic migraineurs and revisiting of friedman’s diagnostic criteria

**DOI:** 10.1038/s41598-025-14758-7

**Published:** 2025-08-22

**Authors:** Islam El Malky, Wael Elshazly Aita, Hazem Abdelkhalek, Amr M. Tayel, Mahmoud Abdelhafiz

**Affiliations:** 1 Associate Prof. of Neurology, Department of Neurology, Director of Interventional Neurovascular Unit, South Valley University, Qena, Egypt; 2 MD Ophthalmology, Department of Ophthalmology, South Valley University, Qena, Egypt; 3 Assistant Professor of Neurology, Department of Neurology, Tanta University, Tanta, Egypt; 4 MD Neurosurgery, Department of Neurosurgery, South Valley University, Qena, Egypt; 5 MD Neurology, Department of Neurology, South Valley University, Qena, Egypt

**Keywords:** Neurology, Pathogenesis, Risk factors

## Abstract

The application of Friedman’s criteria to diagnose suggested IIH WOP will prevent many chronic patients with migraine (CM) from proper diagnosis and treatment. Our prospective study aimed to compare the prevalence of suggested IIH WOP in case of following Friedman’s criteria and in case of novel proposed criteria (OP > 200 mmH2O and radiological finding ≥ two), also reporting the predictive radiological signs for IIH WOP. Refractory chronic patients with migraine underwent ophthalmologic, neurological evaluation, MRI, and a lumbar puncture (LP) with opening pressure (OP) measurement. CSF withdrawal was performed in patients with CSF OP > 200 mmH20. Suggested IIHWOP was defined according to Friedman’s criteria and our novel criteria. Regression analysis was performed to detect the strongest predictor of suggested IIHWOP. The effect of CSF withdrawal was evaluated clinically after two months. One hundred and two consecutive CM were enrolled (95 F, age 32.34 ± 9.45, and BMI 29.04 ± 5.89) without papilledema. Eighteen patients (17.65%) had OP greater than 250 mmH2O, and 20 patients (19.61%) with OP ≥ 200 mmH2O and ≤ 250 mH2O. Prevalence of suggested IIH WOP, according to Friedman’s diagnostic criteria, was three patients (2.9%) only. In case of our novel diagnostic criteria (Absent 6th nerve palsy, ICP > 200 mmH2O, and ≥ two radiological signs), eight patients (7.8%) were discovered. After CSF withdrawal, 85% of patients with migraine with OP > 200 mm H2O improved, specially CM with bilateral Transverse sinus stenosis (TSS). The prevalence of suggested IIH WOP in CM was 7.8%. Bilateral TSS was the only predictor of IIHWOP.

## Introduction

Migraine is a neurological disorder that affects many people across the world and can cause severe disability during the attacks. Chronic migraine can be frustrating to the patient and the neurologist because management is usually difficult and not satisfactory. An interesting discussion on this topic may be the frequent co-occurrences of migraine and idiopathic intracranial hypertension (IIH) suggesting some pathophysiological links between these two conditions^1,2^. Idiopathic intracranial hypertension can be defined as a disease constituting of symptoms developing due to increased intracranial pressure which is not associated with any detectable cause. Remarkably, several reports have drawn attention to a subtype of IIH without papilledema (IIH WOP), and the diagnosis of which may constitute strictly a challenging issue^3,4^. The prevalence of IIH WOP on the general population is not known currently as there is no prevalence study on this entity, in particular that lumbar puncture is not a routine investigation in chronic refractory headache^5,6^. However, recent studies have emerged non papilloedemic IIH as a condition to be closely linked with migraine pathophysiology and the experts remarked non papilloedemic IIH as a possible new risk factor for migraine chronification^1,3^. Also, false negative results could be due to increased CSF opening pressure could be occurring in cases like anxiety, pain, coughing, crying and neck flexion. Also, false negative results such as underlying hypermobile disorders in which the opening pressure of IIH patients is lower than expected in addition to the possibility of diurnal fluctuation of CSF measurement, so it is like “a snap shot”^7^. Also, the normal upper limit for opening pressure of LP might be different between normal population with different gender and body mass index^8^. Diagnosis of IIH WOP was determined by elevated CSF pressure (> 250 mm H2O), in addition to one of these two objective findings (abducent nerve and/or a MRI neuroimaging criteria for definite IIH WOP or suggested IIH WOP, respectively (revised Friedman criteria)^9^. The neuroimaging criteria is the presence of three out of four radiological findings (pituitary empty, distended optic sheath diameter, sinus stenosis and flat posterior globe). In clinical practice, sixth nerve palsy, presence of elevated CSF pressure more than 250 mmH2O or presence of full radiological criteria (three out of four) were difficult to present in chronic patients with migraine, in addition to underlying IIH WOP, making this diagnostic criterion less sensitive especially in this specific population. The application of these criteria will prevent many patients from proper diagnosis and treatment. Our prospective study aimed to compare the prevalence of suggested IIH WOP following the revised Friedman criteria for IIH WOP and when we apply a novel proposed criterion for IIH WOP (Absent Abduscent palsy, OP > 200 mmH2O and radiological finding ≥ two). Also we would like to discover the strongest predictive radiological sign for IIH WOP.

## Methods

### Participants, inclusion and exclusion criteria

A prospective cohort study was conducted. All cases of chronic migraine (CM) were collected prospectively from October 2023 to April 2024 in our neurology clinic, according to (CM) definition by the International Classification of Headache Disorders 3th edition^10^. The study was approved by our institute (ethical approval number: SVU/MED/NAP020/4/23/11/755) and according to Helsinki declaration. Informed consent was taken from all participants. We included patients > 18 years with CM, not responding to treatment. Decision of unresponsive headache to treatment was taken after two preventive medications for two consecutive months. Any medications could have any effect on CSF measuring such as retinoids, topiramate and indomethacin were stopped for at least five and a half drug half-lives for washing out^11^. We excluded all CM patients with papilledema, other causes of secondary headache (infection, tumor and sinus thrombosis), less than 18 years old and with general medical causes of headache.

Neurological and ophthalmological examination were done. Demographic and Clinical data were collected such as age, sex, marital status, having children, symptoms (tinnitus, blurred vision, diplopia, aura), family history, duration of headache complain and body mass index. We collected diary headache data (frequency and severity) two months before (as baseline data) and after lumbar puncture (LP) to avoid the confounding effect of post-lumbar puncture headache (PLPH). Improvement after aspiration was considered if the severity of headache or days of headache per month decreased to less than 50% of its previous level before aspiration.

### Study protocol

MRI-brain and MR venography was performed to all patients, using 1.5 T closed MR Imager using standard surface coil. Five MR sequences were done, Sagittal T1WI, Axial DWI, Axial T2w, FLAIR (fluid-attenuated inversion recovery), MR venography with contrast, and Coronal STIR (fat-suppressed inversion recovery sequence), with special cuts on orbit. Collected data from MRI were the presence of bilateral transverse sinus stenosis, or alternatively, unilateral with contralateral hypoplasia. Stenosis was 50%reduction in lumendiameter. Also, pituitary height (empty if height less than 4.8 mm), optic nerve sheath diameter (considered large if average of both sides more than 5.5 mm), and flattening of the posterior surface of the globe were reported.

All patients had LP in lateral recumbent position with extended neck and stretched, relaxed legs without any straining to avoid increasing of CSF pressure, under complete aseptic condition. Aspiration was performed for cases with OP more than 200 mmH20 until normalization and only 5 mm was used for analysis to exclude infection. We divided the cases into three groups. Group (1) represents cases with OP < 200 mmH2O, group (2) represents cases with OP ≥ 200 mmH2O and ≤ 250 mmH2O, and group (3) represents cases with OP > 250 mmH2O. We also compared the results between patients with the novel proposed IIHWOP criteria and patients with OP ≥ 200 mmH2O but with radiological findings less than two.

### Statistical analysis

**Fig. 1:**
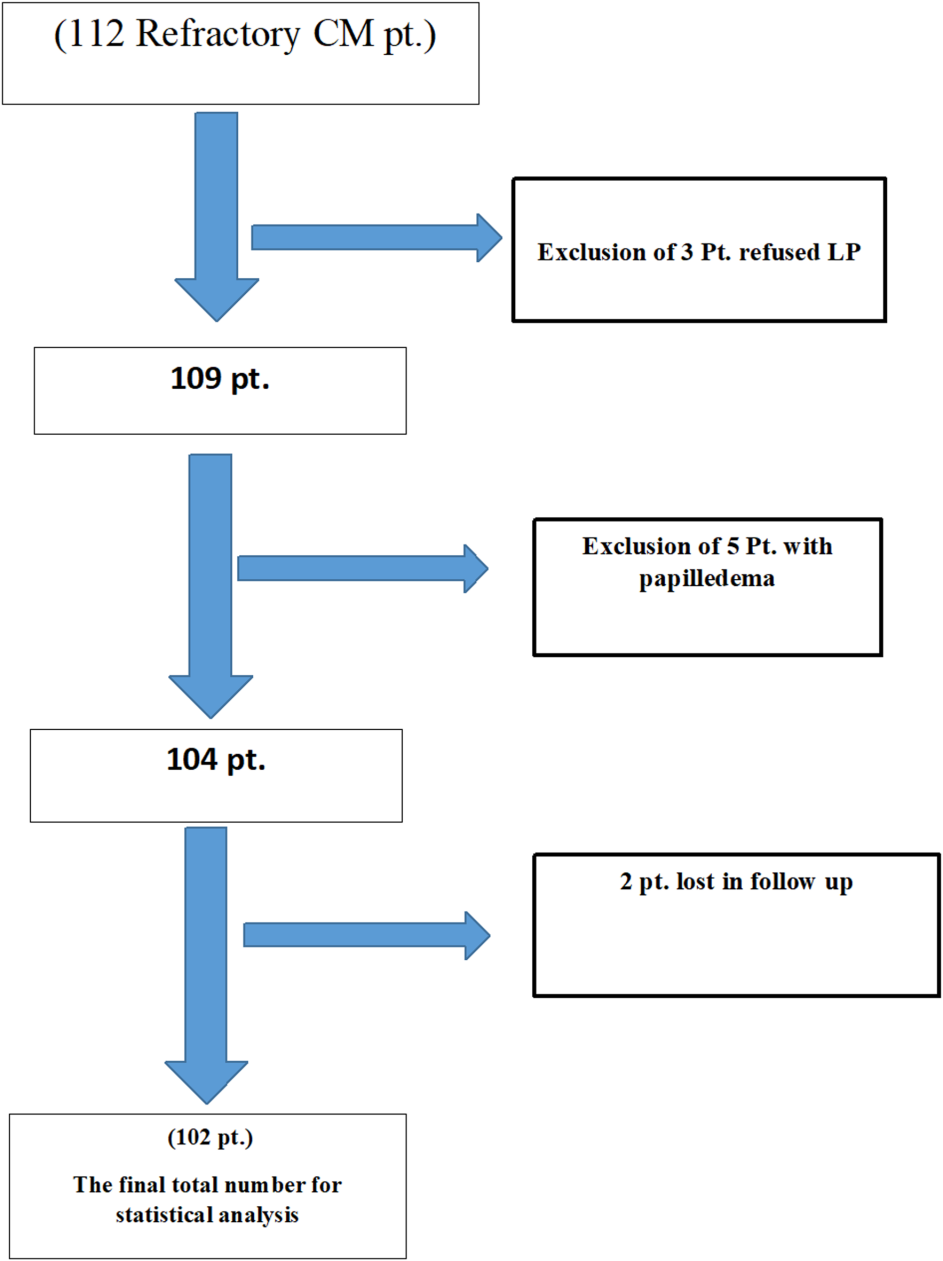
Flow chart.

All clinical and radiological data were collected and analyzed by SPSS software package (version 28). Sample size was calculated according to the expected frequency of IIH WOP which was not more than 10% in CM population according to multiple previous studies which used revised Friedman criteria for IIH WOP^5,6^. The population was at that time 3,600,000 in our governorate. The worldwide migraine prevalence is 14–15%^12^. The result of calculation was 98 participants with confidence limits 5% and confidence level 90%. The final actual number of participants was 102 (Fig. 1).

Continuous variables are expressed as mean ± standard deviation. The variables were assessed by the Kolmogorov Smirnov test for normality distribution. Inter-group analyses were performed with Student’s t test or ANOVA for normally distributed variables and the Mann-Whitney U test for variables which was not normally distributed. The chi square test was used for the comparison of qualitative data. Multivariate binary regression analysis model was performed between clinical and MRI findings to investigate the independent predictors for the presence of IIH WOP.

## Results

### Demographic data

One hundred and two patients were collected to our study. Ninety-five Patients (93.14%) were female with mean BMI 29.04 ± 5.89 and mean age 32.34 ± 9.45. Median Chronification time of headache complain was 3 years (IQR: 1.8-6). There was no any remarkable neurological or ophthalmological finding.

LP revealed 64 patients (62.75%) with OP < 200 mmH2O (group 1) while 20 patients (19.61%) with OP ≥ 200 mmH2O and ≤ 250 mH2O (group 2). Eighteen patients (17.65%) had OP > 250 mmH2O (group3). Demographic data and LP OP (lumber puncture opening pressure) of the three groups were reported in (Table 1). We noticed that there was no statistically significant difference between all groups as regarding age, sex, BMI, and other clinical manifestations while there was a statistically significant difference in LP OP between the groups with p value = 0.001.

**Table 1:** The demographic data between the three groups.

Variable	Group 1 ICP < 200 mmH2O	Group 2 250 ≤ ICP ≥ 200 mmH2O	Group 3 ICP > 250 mmH2O	*P* value
BMI (Mean ± SD)	28.6 ± 5.95	29.4 ± 5.80	30.1 ± 5.93	0.602
Age (Mean ± SD)	31.52 ± 8.97	33.83 ± 9.76	33.61 ± 10.98	o.521
Sex N(%)	60 (93.8%)	19(95%)	16(88.9%)	0.561
Marital state (single) N(%)	8 (12.5%)	4 (20%)	3 (16.7%)	0.526
Having Kids N(%)	54 (84.4%)	14(70%)	15(83.3%)	0.608
Family history N(%)	7(10.9%)	0 (0%)	4(22.2%)	0.422
Blurred vision N(%)	2 (3.1%)	0 (0%)	1 (5.6%)	0.791
tinnitus N(%)	0 (0%)	1 (5%)	0 (0%)	0.561
Allodynia N(%)	11 (17. 2%)	6 (30%)	5 (27.8%)	0.225
Fibromyalgia N(%)	10 (15.6%)	6 (30%)	5 (27.8%)	0.160
Years of complain Median (IQR)	3.5 (0.3–40)	3.5 (0.3–17)	3 (0.6–20)	0.884
Presence of Aura N(%)	6 (9.4%)	7 (35%)	2 (11.1%)	0.321
LP OP	173.4 ± 19.3	219 ± 8.5	338.6 ± 75.2	0.001*

ICP: Intracranial Pressure, LP OP: lumbar puncture opening pressure, N: number, IQR: Interquartile Range, BMI: Body Mass Index.

(*): Statistically significant.

No abnormality was found in MRI of all patients except six patients with only two radiological signs of increased ICP and five patients with three radiological signs. According to Friedman criteria (Absent 6th nerve palsy, ICP > 250 mmH2O and ≥ three radiological signs), prevalence of suggested IIH WOP was applied only in three patients (2.9%). Conversely, by our newly proposed criteria (Absent 6th nerve palsy, ICP > 200 mmH2O and ≥ two radiological signs), more five patients were added to IIH WOP and the prevalence became 7.8% (8 patients). We reported the demographic data between the novel proposed IIH WOP and cases with OP > 200 mmH2O and radiological signs < two in (Table 2). There was no statistically significant difference.

**Table 2:** The demographic data between cases with novel proposed criteria of IIHWOP and cases with ICP > 200 mmH2O with < 2 radiological signs.

Variables	Novel proposed criteria of IIHWOP (8 patients)	ICP > 200 mmH2O, radiological signs < 2 (30 patients)	*P* value
BMI (Mean ± SD)	28.6 ± 5.95	30.1 ± 5.93	0.602
Age (Mean ± SD)	31.52 ± 8.97	33.61 ± 10.98	0.521
Sex N(%)	8 (100%)	27(90%)	1
Marital state (single) N(%)	8 (100%)	23 (76.7%)	0.307
Having Kids N(%)	8 (100%)	21(70%)	0.159
Family history N(%)	1(12.5%)	3(10%)	1
Blurred vision N(%)	1(12.5%)	0 (0%)	0.211
tinnitus N(%)	0 (0%)	1 (3.3%)	1
Allodynia N(%)	2 (25%)	9 (30%)	1
Fibromyalgia N(%)	2 (25%)	9 (30%)	1
Years of complain Median (range)	3.5 (0.3–40)	3 (0.6–20)	0.884
Presence of Aura N(%)	3 (37.5%)	6 (20%)	0.363
LP OP: Mean ± SD	26.40 ± 7.481	31.38 ± 8.40	0.112

LP OP: lumbar puncture opening pressure, N: number, IQR: Interquartile Range, BMI: Body Mass Index.

### Radiological data

Radiological signs of MRI were reported in (Table 3) in the novel proposed IIHWOP patients and other cases with increased ICP > 200 mmH2O and MRI radiological signs less than two. There was a significant difference at all MRI radiological finding between both groups. All statistically significant MRI radiological findings between both groups were used in multivariate binary regression model. We found that bilateral TSS (bilateral stenosis or unilateral stenosis plus transverse sinus hypoplasia) is the only predictor for IIH WOP diagnosis with p value = 0.001 [95% CI (2.9–10.7)].

**Table 3:** MRI radiological finding between proposed IIHWOP and cases with ICP > 200 mmH2O with < 2 radiological signs.

Radiological finding	Novel proposed criteria of IIHWOP (8 patients)	cases with ICP > 20 mmH2O with < 2 radiological signs (30 patients)	*p*-value
flattening of the posterior ocular globe TSS Rt ONSD by MRI Median (IQR) Lt ONSD by MRI Median (IQR) Pituitary empty N (%)	3(37.5%) 5(62.5%) 4.5(4.2–5.1) 4.5(4.1–4.5) 6(75%)	0(0%) 5(16.7%) 4.3(3.8-5) 4.2(3.7–4.2) 2(6.7%)	0.007* 0.019* 0.001* 0.001* 0.001*

TSS: Transverse Sinus Stenosis, ONSD: Optic Nerve Sheath Diameter.

(*): Statistically significant.

### Post-CSF aspiration assessment

We assessed the headache after aspiration two months later. Sixty-five patients (63.7%) revealed PLPH within few hours to seven days after aspiration which was relieved by bed rest, fluids and NSAIDs (non-steroidal anti-inflammatory drugs). Headache improvement occurred in group (3), in addition to group (2) in comparison to group (1) with statistically significant difference (88.9%,85%, 50% respectively with p value 0.001).

There was no statistical difference between IIH WOP cases with the novel proposed criteria and other cases with increased ICP > 200 mmH2O and MRI radiological signs < two [(6/8)75% against (27/30) 90%, respectively with p value = 0.271)

We noticed that improvement in cases with bilateral TSS (Transverse sinus stenosis) was higher than in cases without sinus stenosis (92.3%, p value = 0.029), compared with the other radiological signs such as empty pituitary sella turcica (76.9%, p value = 0.366), ONSD (71.4%, p value = 0.564) and flattened globe (83.3%, p value = 0.413).

## Discussion

Our study investigated how the current criteria for diagnosis of IIHWOP (Friedman 2013) actually might lack sensitivity. Using an opening pressure of 200 mmH2O and at least two out of four MRI signs of IIH, we have found more IIHWOP patients in CM (7.8% instead of 2.9%), helping to give a proper diagnosis as well as treatment to those patients. CSF withdrawal to these patients resulted in therapeutic benefit in approximately 85% of the patients.

Our study didn’t find any clinical difference between different CSF pressure groups, especially as regarding sex and BMI. This confirms the similar pathophysiological mechanism between migraine and IIH in previous studies^1,2^. The relation between BMI and both migraine and IIH is well known in different studies which are in concordance with our findings^13^. Favoni et al. found out that IIH cases had BMI higher than in migraine cases without IIH which was opposite to our study^14^.

Our study reported low prevalence of suggested IIH WOP (2.9%) according to revised Friedman criteria^9^, similar to Favoni et al. study because of the similar population chronic migraineurs, unresponsive to preventive treatment. De Simone et al. reported a prevalence of 86.4% and 43.2% in case with OP > 200 mmH2O or > 25 mmH2O, respectively in CM patients which is higher than our study^3^. This discrepancy might be due to selection of CM cases with one radiological finding which was unilateral or bilateral TSS in 72.2% of cases, in addition to CM patients with medication overuse headache (MOH).

The application of revised Friedman criteria might lead to loss of patients with IIH WOP, then proper treatment. We presented novel diagnostic criteria for suggested IIH WOP diagnosis in CM patients with refractoriness. We found that the prevalence raised up to 7.8%. Favoni et al. reported prevalence of IIH WOP 5%, in case of OP > 200 mmH2O with three radiological signs^14^. We decreased OP as a condition for suggested IIH WOP diagnosis because of three reasons. False negative results of decreased CSF opening pressure could be occurring in cases like underlying hypermobile disorders in which the opening pressure of IIH patients is lower than expected^15^, which is in line to collapsibility of dural sinuses is increasingly proposed as a driving mechanism of IIWHOP^16^, in addition to the possibility of diurnal fluctuation of CSF measurement^7^. Also, the normal upper limit for opening pressure of LP might be different between normal population with different gender and body mass index^8^. Third reason was that possibility of OP might be less in IIH WOP than IIH. Also, we suggested ≥ two radiological signs to increase sensitivity of the diagnostic criteria to pick up more cases which might gain benefit from different IIH treatment models such as CSF withdrawal.

We reported the importance of TSS as a predictor for IIH WOP diagnosis. Many studies reported its importance in IIH and IIH WOP diagnosis^17–19^. We found that improvement after CSF withdrawal was higher in cases with TSS than cases without TSS. De Simone et al. used TSS as a single radiological finding in CM patients selection, leading to discover of more CM patients with IIH WOP as mentioned previously^3^.

We noticed improvement of headache after CSF withdrawal even in cases with normal OP which may indicate intermittent elevation of ICP in CM patients, leading to chronicity^7^. Improvement of headache after CSF withdrawal was noticed in cases with more OP (> 250 mmH2O) and or patients with TSS. These findings confirm the importance of CSF OP rather than number of radiological signs. Tables 2 and 3 showed the demographic similarity between both groups (newly proposed criteria group and group with increased ICP > 200 mmH2O and MRI radiological signs less than two) and the different radiological findings, in addition to the headache improvement in both groups, all of these were an evidence of the special importance of TSS in comparison to other radiological findings, regarding improvement. Headache improvement (nearly 50%) in group (1) with OP less than 200 mmH2O might support the hypothesis that a deranged intracranial pressure control, although not sufficient to determine IIH/IIHWOP, may be one of the mechanisms implicated in the pathophysiology of migraine^20^. This finding needs more future studies.

Brief follow up of CSF withdrawal effect in clinical improvement is one of the limitation of our study. We suggest more RCTs for the effect of CSF withdrawal in refractory CM patients.

## Conclusion

Refractory CM patients should be investigated for IIH WOP diagnosis to offer proper plan for treatment. Prevalence of IIH WOP with the proposed novel diagnostic criteria increased to 7.8% in comparison to 2.9% in case of revised Friedman’s Criteria. TSS is the only radiological predictor for IIH WOP.

## Data Availability

The datasets used and/or analyzed during the current study available from the corresponding author on reasonable request.

## Abbreviations

ICP: (Intracranial pressure)
IIH: (Idiopathic Intracranial Hypertension)
IIH WOP: (IIH without papilledema)
ONSD: (Optic Nerve Sheath Diameter)
BMI: (Body Mass Index)
CM: (chronic migraine)
LP: (lumber puncture)
PLPH: (post-lumber puncture headache)
TSS: (Transverse sinus stenosis)
MOH: (medication overuse headache)
